# Acupuncture treatment for chemotherapy-induced nausea and vomiting: A protocol for systematic review and meta-analysis

**DOI:** 10.1097/MD.0000000000020150

**Published:** 2020-05-22

**Authors:** Yueping Huang, Rui Zhang, Qin Yao, Jinyi Liu, Xiali OuYang, Xin Hui, Hao Wang, Rui He, Baixiao Zhao

**Affiliations:** a School of Acupuncture-Moxibustion and Tuina; b School of Traditional Chinese Medicine, Beijing University of Chinese Medicine, Beijing, China.

**Keywords:** acupuncture, chemotherapy, nausea, systematic review, vomiting

## Abstract

**Background:**

Chemotherapy-induced nausea and vomiting (CINV) make many patients have negative adherence of treatment. Acupuncture has been widely applied in treatment of CINV, but its efficacy has not been evaluated scientifically and systematically in recent years. Hence, evaluating the safety and effectiveness of acupuncture treatment in patients with CINV is the main purpose of this review.

**Methods and analysis:**

We will search the following electronic databases from inception to Mar 2020: the Cochrane Central Register of Controlled Trials, EMBASE, PubMed, the Web of Science, China National Knowledge Infrastructure (CNKI), Traditional Chinese Medicine, Chinese Biomedical Literature Database (CBM), Wan-Fang Database and Chinese Scientific Journal Database (VIP database). All published academic data about clinical randomized controlled trials in English or Chinese related to acupuncture for treating CINV will be obtained. The primary outcomes are defined as frequency and severity of CINV during chemotherapy. The secondary outcomes are defined as any adverse events and quality of life of CINV during chemotherapy. The study selection, data extraction, and assessment of study quality will be conducted by 2 researchers independently. Review Manager Software (RevMan) V.5.3 will be performed for meta-analysis.

**Results:**

The results of this review will provide a high-quality synthesis of current available evidence to evaluate acupuncture is an effective and safe treatment for CINV.

**Conclusion:**

The conclusion of this review will provide the highest level of evidence to judge whether acupuncture is an effective and safe treatment for patients suffered from CINV.

**PROSPERO registration number:**

CRD42020153993.

## 1 Introduction

Chemotherapy-induced nausea and vomiting (CINV) are considered the most severe and most distressing among cancer patients.^[1]^ Nausea and vomiting can lead to many undesirable events such as malnutrition, depression, dehydration, and electrolyte disorder.^[2,3]^ Moreover, without valid prevention and control of CINV can affect patients’ quality of life or treatment outcomes.^[4]^ Occasionally, cancer patients are unwilling to continue chemotherapy due to the mentioned reasons. Hence, it is very necessary to find a way of increasing patients’ compliance and decreasing the side effects of chemotherapy in maximum.

There are some antiemetic drugs may play an important role in preventing CINV, including 5-HT3 serotonin receptor antagonists, tachykinin NK1 receptor antagonist, steroids, olanzapine, dopamine receptor antagonists, and benzodiazepines.^[5]^ Although antiemetic drugs can improve symptoms to a certain extent, the high cost of these agents and their side effects such as hypotension, diarrhea, fatigue, and headache, have limited the use of these antiemetic drugs.^[6]^

Acupuncture, as an important part of traditional Chinese medicine (TCM) and complementary medicine, has been extensively used for thousands of years and applied in various diseases in China, it also gets more and more popular all over the world.^[7]^ Acupuncture can stimulate the body to release natural endogenous opioids (endorphins) and neurotransmitters or neurohormones, which can change the uncomfortable experience such as CINV and pain.^[8,9]^ A plenty of high-quality clinical trials have been conducted to prove acupuncture therapy is effective and safe in spite of the mechanism is not completely clear yet.^[10]^ What is more, compared with antiemetic, acupuncture therapy is safe medical procedures with minimal side effects for CINV. The National Institutes of Health (NIH) Consensus Statement has recommended acupuncture therapy as an effective and complementary intervention to prevent CINV.^[11]^

As CINV has been a common problem for patients under chemotherapy, it is necessary to make a systematic review to provide a convincing conclusion whether acupuncture is an appropriate method to treat CINV. Hence, this systematic review adopts the methods of evidence-based medicine to evaluate the overall safety and effectiveness of acupuncture as a complementary intervention in CINV management, providing convincing conclusions of acupuncture prescription is the further work.

## 2 Methods

### 2.1 Study registration

This systematic review protocol has been registered in the International Prospective Register of Systematic Reviews (PROSPERO) as CRD42020153993. The protocol is conducted complying with the preferred reporting items for systematic reviews and meta-analysis protocols (PRISMA-P) statement guidelines.^[12]^ And the study will follow the PRISMA statement guidelines.^[13]^

### 2.2 Criteria for including studies

#### 2.2.1 Types of studies

All the related RCTs of acupuncture therapy to treat CINV in patients will be included. Summary results of completed and ongoing trials published on clinical trial registration platform will be required.

#### 2.2.2 Types of participants

Patients diagnosed with cancer and CINV were included. There will be no limit for age, gender, ethnic origin, and educational or economic status among patients. Patients with mental illness, acute infections, as well as those with diseases that may cause nausea and vomiting, were excluded.

#### 2.2.3 Types of interventions and comparison

Acupuncture therapy is defined as the stimulation of acupuncture points by needles without limitation on needle material, treatment point selection, operation method, needle retention time, and treatment course, the trials in which patients were treated with other stimulating methods, such as acupressure, moxibustion, acupoint injection, laser acupuncture, and cupping will be excluded.

Control interventions involving sham acupuncture, placebo, usual care, medication, no treatment, and other conventional therapies will also be included.^[14]^ Studies comparing the efficacy of different acupoint prescriptions or other complementary and alternative therapeutic intervention (e.g., Chinese herbal medicine) will be excluded.

#### 2.2.4 Types of outcome measures

##### 2.2.4.1 Primary outcomes measures

The primary outcomes will be defined as severity and frequency of CINV. Scales or medical or nursing observations will be accepted as the measurement.

##### 2.2.4.2 Secondary outcomes measures

The secondary outcomes will be any adverse events and quality of life of CINV and measured by validated scales.

### 2.3 Search strategy

#### 2.3.1 Electronic searches

The following electronic databases will be searched from inception to Mar 2020: English databases such as the Cochrane Central Register of Controlled Trials, EMBASE, PubMed, the Web of Science, and Chinese databases such as China National Knowledge Infrastructure, Traditional Chinese Medicine, Chinese Biomedical Literature Database (CBM), Wan-Fang Database, and Chinese Scientific Journal Database (VIP database). All published academic data about clinical randomized controlled trials in English or Chinese related to acupuncture for treating CINV will be obtained.

The following search terms will be used: chemotherapy, nausea, vomiting, emesis, acupuncture, manual acupuncture, filiform steel needle, electroacupuncture, fire needling, auricular acupuncture, ear acupuncture, dermal needle, abdominal acupuncture, pyonex, and plum blossom needle. The equivalent search words will be used in the Chinese databases. The search strategy for the other online databases was adjusted according to their requirements.

The search strategy for PubMed is shown in Table 1.

**Table 1:**
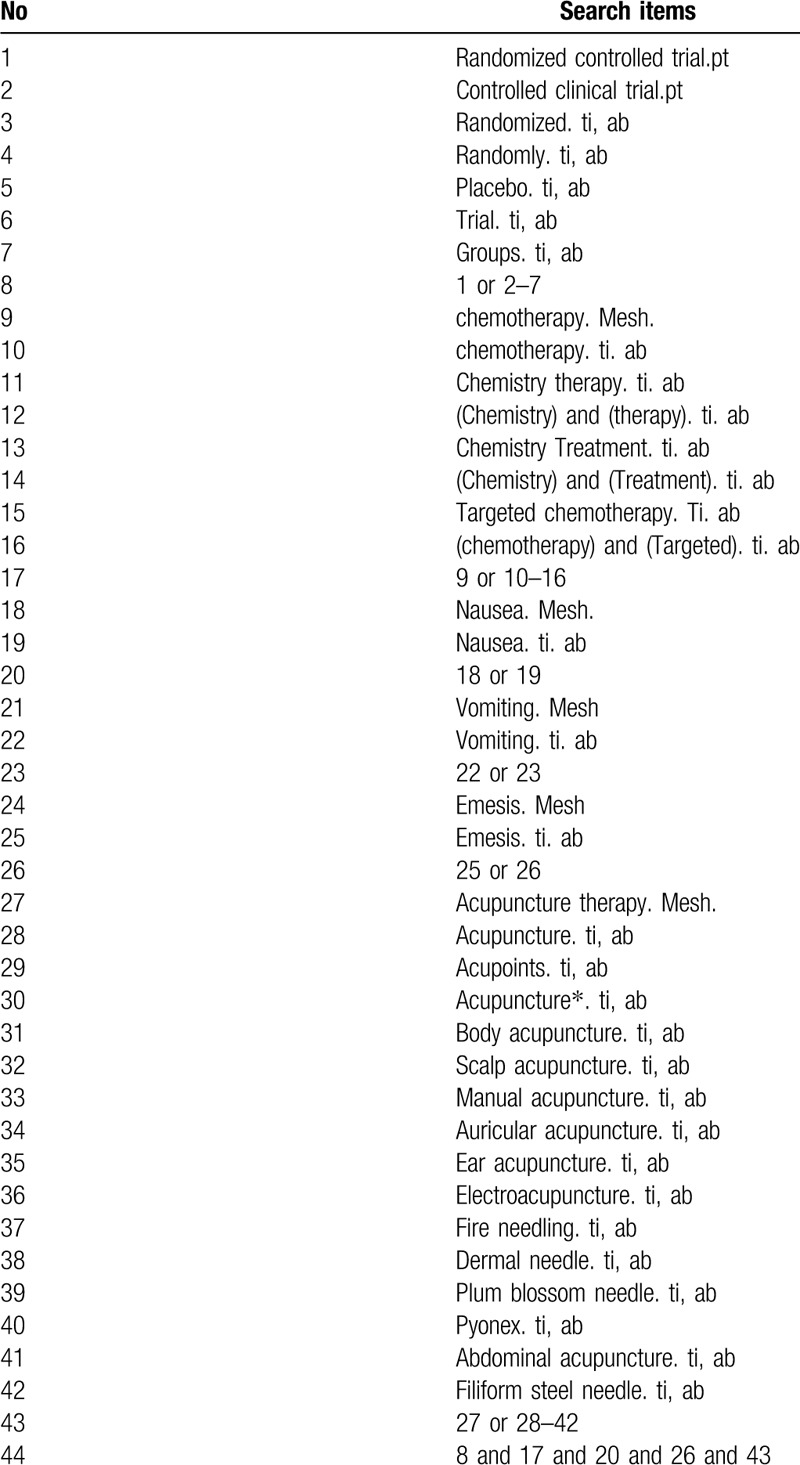
Search strategy used in PubMed.

#### 2.3.2 Searching other resources

We will search for additional trials by examining the reference lists of studies related to acupuncture therapy for CINV. Ongoing or unpublished trials will be search from the NIH Clinical Trails, the International Clinical Trials Registry Platform, and the Chinese Clinical Register.

### 2.4 Data collection and analysis

#### 2.4.1 Selection of studies

**Figure 1:**
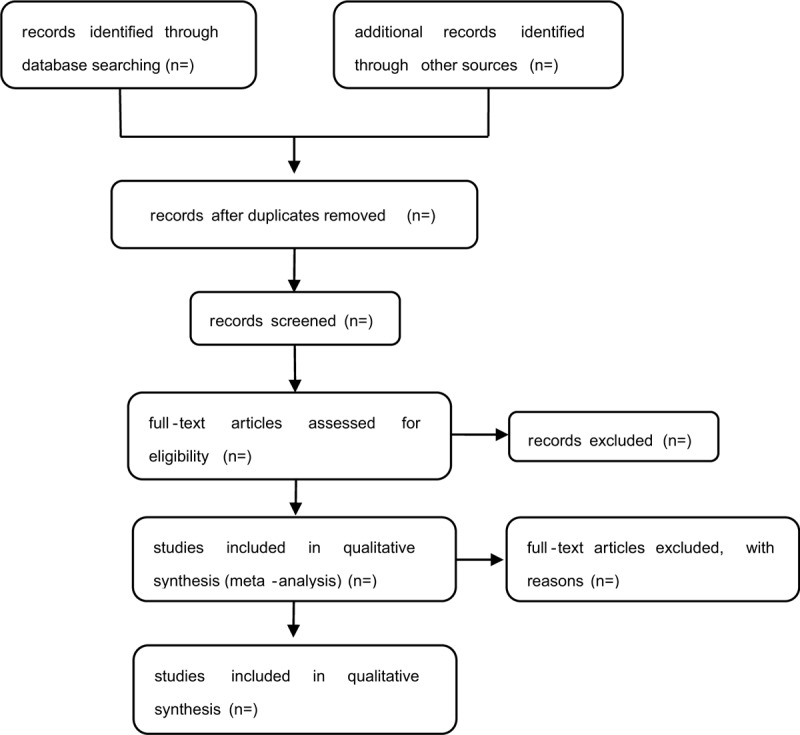
Study flow diagram.

The studies selected from the databases were integrated into Endnote software (X9.2.). After omitting duplicate studies, 2 authors (YPH and RZ) independently conducted primary screening by checking the titles and abstracts according to the inclusion criteria. Then, 2 reviewers subsequently will obtain full text reports for further assessment. Differences in opinion between the 2 authors were arbitrated by a third author (BXZ). We will contact authors of trials for clarification when necessary. The study flow diagram is shown in Figure 1.

#### 2.4.2 Data extraction and management

The data from each included trial were extracted and recorded in a data extraction form by 2 independent authors (YPH and RZ). The following factors were analyzed: general information (country of publication, year of publication, language, funding, and author details), participants (age, baseline characteristics, disease duration, inclusion criteria, exclusion criteria, and sample size), interventions (type of acupuncture/control, treatment frequency, treatment duration, and course of acupuncture), outcomes (primary and secondary outcomes, method of outcome assessments). A third reviewer will be set to be discussed with and judge the disagreements during the course. The authors of the studies were contacted, when required. Review Manager Software (RevMan) V.5.3 will be used for data analysis and synthesis.

#### 2.4.3 Assessment of risk of bias in included studies

The Cochrane Collaboration tool for assessing the risk of bias in randomized trials was used to evaluate the methodological quality in each included trial.^[15]^ The risk of bias evaluations were performed by 2 authors (JYL and XLOY) independently: selection bias (random sequence generation and allocation concealment), performance bias (blinding of the participants), detection bias (blinding of the outcome assessment), attrition bias (incomplete outcome data), selective outcome reporting bias, and other sources of bias. The risk of bias will be evaluated as low, high, or unclear.

#### 2.4.4 Measures of treatment effect

We will use RevMan V.5.3 for data analysis and quantitative data synthesis. Mean difference (MD) or standard MD (SMD) will be used to express the continuous data, while risk ratio (RR) will express the dichotomous data. The estimated value and 95% confidence interval (95% CI) of each effect size will be given.

#### 2.4.5 Unit of analysis issues

The individual participant will be used as the analytical unit. We will not include cluster-randomized trials and crossover studies because they lack the appropriate design for the study objectives.

#### 2.4.6 Management of missing data

We will attempt to obtain the missing or incomplete data by contacting the author of the articles. If the data cannot be provided, we will explain the situation and use the available data to accomplish our analysis.

#### 2.4.7 Assessment of heterogeneity

*χ*^2^ test will be used to calculate the heterogeneity of the research results and, *I*^2^ statistic test will be used to quantify inconsistency. If the *I*^2^ is less than 50%, we will use the fixed-effect model. Otherwise, the random effect model and descriptive analysis will be considered.

#### 2.4.8 Assessment of reporting biases

Funnel plots will be used to evaluate the reporting biases if the numbers of studies included in the meta-analysis are sufficient more than 10 studies.^[16]^

#### 2.4.9 Data synthesis

If the meta-analysis is possible for this review, RevMan V.5.3 software will be used in data synthesis. We will express dichotomous data in RR and continuous data in MD or SMD. The fixed-effects model will be put into use if *I*^2^ < 50% and *I*^2^ > 75%, *I*^2^ < 50% will be classified as having low heterogeneity, whereas those with *I*^2^ > 75% will be classified as having high heterogeneity. We will then offer a descriptive analysis or subgroup analysis.

#### 2.4.10 Subgroup analysis

Subgroup analysis will be conducted if the data are sufficient, according to the differences in acupuncture methods, patient conditions, and control.

#### 2.4.11 Sensitivity analysis

Sensitivity analysis will be performed to evaluate the impact of sample size, study design, methodological quality and the effect of missing data, and to verify the robustness of the primary decisions of the review conclusions. The meta-analysis will be repeated after low-quality studies are excluded.

#### 2.4.12 Grading the quality of evidence

The quality of evidence for all outcomes will be evaluated by the grading of the Grading of Recommendations Assessment, Development, and Evaluation will be the tool to evaluate the quality of the evidence.^[17]^ Risk of bias, inconsistency of results, indirectness, imprecision, and publication bias will be considered. The assessments will be categorized as very low, low, moderate, or high.^[18]^

## 3 Discussion

Acupuncture therapy not only has the characteristics of higher acceptability, but also less side effect and inexpensiveness. Acupuncture could effectively reduce the symptoms of CINV.^[19,20]^ Acupuncture therapy could be recommended as a non-pharmacological method applied to patients with CINV. But their overall efficacy and safety have not been evaluated scientifically and systematically in recent years. Hence, our study systematically will assess the efficacy and safety of acupuncture for CINV. We will divide the review into 4 sections including identification, study inclusion, data extraction, and data synthesis. This review will help the clinical doctors to choose acupuncture as an alternative treatment for CINV patients, and offer the patients more options to relieve their symptoms.

## Author contributions

YPH and RZ conceived and designed the study, this manuscript was mainly completed by them and they contributed equally to this work and are co-first authors. The manuscript of the protocol was drafted by YPH, RZ and revised by BXZ. YPH and RZ will independently screen the potential studies and extract data from the included studies. JYL and XLOY will assess the risk of bias and finish data synthesis. BXZ will arbitrate any disagreements and ensure that no errors occur during the review. All authors read, provided feedback, and approved the final manuscript.

**Data curation:** Jinyi Liu, Xiali OuYang.

**Funding acquisition:** Baixiao Zhao.

**Investigation:** Rui He.

**Methodology:** Rui He.

**Software:** Xin Hui, Hao Wang.

**Supervision:** Baixiao Zhao.

**Visualization:** Jinyi Liu, Xiali OuYang, Qin Yao.

**Writing – original draft:** Yueping Huang, Rui Zhang.

**Writing – review and editing:** Yueping Huang, Rui Zhang, Qin Yao, Baixiao Zhao.
